# Reactive Liftoff of Crystalline Cellulose Particles

**DOI:** 10.1038/srep11238

**Published:** 2015-06-09

**Authors:** Andrew R. Teixeira, Christoph Krumm, Katherine P. Vinter, Alex D. Paulsen, Cheng Zhu, Saurabh Maduskar, Kristeen E. Joseph, Katharine Greco, Michael Stelatto, Eric Davis, Brendon Vincent, Richard Hermann, Wieslaw Suszynski, Lanny D. Schmidt, Wei Fan, Jonathan P. Rothstein, Paul J. Dauenhauer

**Affiliations:** 1 University of Massachusetts Amherst, Departments of Chemical and Mechanical Engineering, Amherst, MA, 01375; 2 University of Minnesota, Department of Chemical Engineering and Materials Science, Minneapolis, MN, 55455, USA; 3 Catalysis Center for Energy Innovation, a U.S. Department of Energy – Energy Frontiers Research Center. University of Delaware, Newark, DE, 19716, USA

## Abstract

The condition of heat transfer to lignocellulosic biomass particles during thermal processing at high temperature (>400 °C) dramatically alters the yield and quality of renewable energy and fuels. In this work, crystalline cellulose particles were discovered to lift off heated surfaces by high speed photography similar to the Leidenfrost effect in hot, volatile liquids. Order of magnitude variation in heat transfer rates and cellulose particle lifetimes was observed as intermediate liquid cellulose droplets transitioned from low temperature wetting (500–600 °C) to fully de-wetted, skittering droplets on polished surfaces (>700 °C). Introduction of macroporosity to the heated surface was shown to completely inhibit the cellulose Leidenfrost effect, providing a tunable design parameter to control particle heat transfer rates in industrial biomass reactors.

A microcrystalline cellulose particle can be analogous to a boiling water droplet on a heated surface. When subjected to high temperatures (>400 °C), long chain biopolymers such as cellulose decompose into smaller, more valuable products used for renewable fuels and chemicals. The chemistry of cellulose decomposition is the subject of ongoing investigation^1^,^2^,^3^,^4^; promotion of the desirable reaction pathways and/or variation of the heating rate can strongly alter product distribution consisting of hundreds of chemicals^5^,^6^. Recent work has shown that long chain, crystalline cellulose reacts to a short-lived liquid intermediate with millisecond lifetime comprised of molten oligomers before decomposing to vapors^7^,^8^,^9^. Despite strong dependence on heat transfer, there is limited understanding of the multi-phase behavior of non-volatile molten cellulose on high temperature surfaces^7^. Here, we show that surface structure and temperature strongly alter the dynamics of pyrolyzing cellulose. The microcrystalline particle evolves into a liquid intermediate droplet and transitions through heat transfer regimes, including the film-boiling Leidenfrost effect, where complete particle liftoff is observed. The resulting heat transfer rates vary by nearly an order of magnitude across a temperature range commonly used for pyrolysis. Surface macropores, such as those found on catalysts, suppress cellulose droplet liftoff and enhance heat transfer to the intermediate liquid. Discovery of the cellulose Leidenfrost effect as a result of reactive vapor production from an initially crystalline solid helps contextualize the importance of heat transfer to solid biopolymers and reveals the capability of structured surfaces for controlling reacting particles.

Design of inorganic surfaces for enhanced heat transfer aims to control the droplet interface, with increased wetting and surface area providing greater contact and transfer of thermal energy. At higher surface temperatures, liquids such as water undergo a transition to Leidenfrost film boiling, where vapor production reaches a critical rate such that a vapor/gas layer forms between the heated surface and the liquid^10^,^11^,^12^,^13^,^14^. The transition to a completely de-wetted state is accompanied by a dramatic reduction in heat transfer into the liquid, greatly increasing the lifetime of the droplet^14^.

In contrast, macromolecules such as cellulose are not known to lift off of high temperature surfaces. Comprised of long chains of six-carbon sugars (10^2^-10^3^ units long), cellulose forms microcrystalline domains in solid particles with sharp edges and macropores. It has previously been shown that, under pyrolytic conditions, cellulose chain size reduction via intra-chain (glycosidic) cleavage forms short-chain oligomers, which melt and form liquid droplets for 100–200 ms before further reacting to volatile species (at most six carbons in size) and evaporating^6^,^15^. However, intermediate liquid cellulose has previously been shown to wet surfaces such as alumina at temperatures as high as 700 °C^7^.

In this work, it is shown through high-speed visualization that crystalline cellulose exhibits the Leidenfrost film boiling effect as a result of reactive gas and vapor production during the millisecond-scale liquid intermediate lifetime. Molten cellulose droplets are observed to lift off of high temperature surfaces (driven by off gas flow) and move erratically. With the introduction of macroporous heated surfaces, complete suppression of the Leidenfrost effect is observed, with product vapors flowing through surface pores. Collapse of the vapor layer suppresses droplet motion due to improved droplet/surface contact and increases the rate of heat transfer from the surface into the particle.

Imaging of reacting particles was performed using combined high-speed photography and microscopy with a temperature-controlled reaction stage. A 90-degree mirror was used for profile visualization, allowing for determination of wetting behavior and contact angles of droplets. Microcrystalline cellulose with a 220 μm average particle diameter was impinged upon the hot stage, and the resulting liquid intermediate evaporation rate was measured for temperatures between 500 °C and 775 °C. Experiments were performed on a silicon surface polished to 5 μm surface roughness as well as silica and alumina particles that were pressed at 15 kpsi and calcined, creating a macroporous surface.

## Results & Discussion

**Figure 1:**
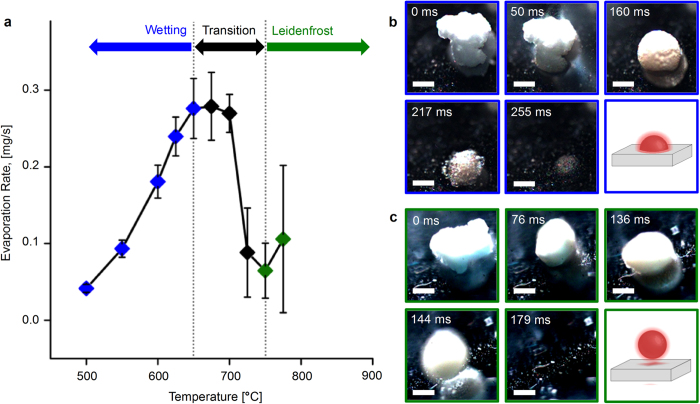
Reactive Liftoff of Crystalline Cellulose Particles on Polished Silicon. **a**. The rate of evaporation of initially crystalline cellulose particles (average 220 μm) varies by an order of magnitude as the intermediate droplet transitions from low temperature wetting (blue) to film boiling (black) and into the Leidenfrost regime (green). Error bars represent 95% confidence. **b**. Initially microcrystalline cellulose forms a melt (160 ms), wets polished silicon at 625 °C, and completely evaporates by 250 ms. See extended data for video. **c**. Microcrystalline cellulose particles form a melt on polished silicon at 750 °C which lifts off the surface and moves out of frame (179 ms). Scale bars = 100 μm.

For cellulose impinging on a polished silicon surface, the solid particle transitions to a liquid droplet, after which the liquid droplet reacts to primarily form gases and vapors. The reactive evaporation rate, which correlates with the rate of heat transfer into the droplet^14^, is defined as the change in mass of the cellulose droplet from the time it is completely liquid until it fully reacts divided by that time interval^†^. For low temperatures (500–650 °C), intermediate liquid evaporation rate increases nearly linearly with temperature (Fig. 1a). In this regime, liquid intermediate cellulose wets the surface, providing rapid solid-liquid heat transfer (Fig. 1b). Receding contact angles for wetting intermediate liquid cellulose were 56–62 degrees^†^. However, between 675 °C and 750 °C, the evaporation rate decreases with increasing temperature. At these temperatures, the rate of vapor and gas production is sufficient such that film boiling was observed, with a gas layer greatly reducing heat transfer between the surface and the particle. Above 750 °C, the intermediate liquid fully de-wets the surface and evaporation rate increases with further increases in temperature. For these conditions, intermediate cellulose liquid moved erratically on polished silicon surfaces.

The heat transfer curve measured in Fig. 1a closely resembles those measured in conventional liquid evaporation Leidenfrost curves^16^. Observed heat transfer rates vary by nearly an order of magnitude as the particle is subject to transitioning modes of heat transfer. At low temperatures, solid-liquid conduction via wetting provides rapid thermal flux into the particle. With the onset of film boiling, convection through the gas layer becomes the dominant mode of heat transfer. This result indicates that, above 650 °C, the lifetime of a reacting cellulose particle is determined by the rate of heat transfer into the particle. Reacting cellulose intermediate liquid differs from conventional Leidenfrost droplet volatile liquids in that the heat flux supplied to the droplet is balanced by both heat of vaporization as well as heat of reaction for cellulose^17^.

The uniqueness of a Leidenfrost cellulose particle undergoing reaction derives from the balance of surface heat transfer and biopolymer reaction rates. Comparison of the timescale for reaction, based on cellulose pyrolysis kinetics^18^,^19^, with the timescale for heat transfer to the droplet via a dimensionless “Reacting Leidenfrost” quantity, ϕ_RL_ = τ_conv/_τ_rxn_ = ρC_p_L_c_k_rxn_/h, provides two extreme conditions^†^. At small ϕ_RL_, negligible reaction implies the heating of solid unreacting particles, whereas at large ϕ_RL_, the particle fully reacts to a slowly evaporating liquid and may exhibit Leidenfrost behavior consistent with conventional liquids (e.g. methanol or water). However, cellulose in the range of 500–700 °C as depicted in Fig. 1 exhibits comparable heat transfer and reaction rates^†^, 10^−1^ < ϕ_RL_ < 10^+1^, such that reaction, evaporation, and particle liftoff occur simultaneously.

**Figure 2:**
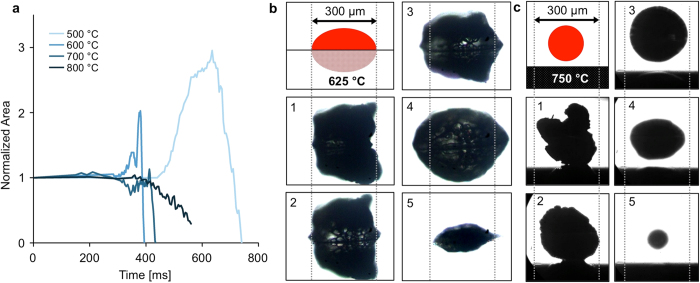
Individual Cellulose Particles on Polished Silicon. **a**. Cross sectional area of cellulose particles (initially ~300 μm) normalized to initial values with reaction time (0–800 ms) for 500–800 °C on polished silicon. **b**. Profile images of particles at lower temperature (625 °C), which liquefy and wet with increased contact area before rapidly evaporating. See extended data for full video. **c**. Profile images of particles at higher temperatures (750 °C), where crystalline cellulose liquefies and off gases at sufficient rate to lift molten cellulose droplets above the surface.

The complexity of particle conversion results in dramatic variation in surface interaction. The apparent cross-sectional cellulose particle area as viewed from above and normalized to the original particle area was traced with time between 500 °C and 800 °C and plotted in Fig. 2a. At 500 °C and 600 °C, the particle area first increases, as the intermediate liquid cellulose spreads to wet the polished silicon surface. This is followed by rapid decrease in area as the intermediate liquid reacts, evaporates and shrinks. At 800 °C – in the Leidenfrost regime – the particle does not spread to wet the surface and shows significantly reduced heat transfer as the particle slowly reacts. Even at 800 °C, the particle exhibits an overall lifetime similar to that observed below 600 °C. Figure 2b,c depict cellulose on a polished surface at 625 °C and 750 °C respectively, showing the qualitative differences between droplet spreading and wetting at lower temperatures and de-wetting in the Leidenfrost regime above 750 °C. Previous studies have shown that the vapor layer underneath a Leidenfrost droplet is very thin (particle radius >> vapor film height) for the majority of the droplet lifetime^20^,^21^. As observed in Fig. 2c, the droplet height is on the same order of magnitude as droplet radius, indicating a substantial rate of gas and vapor production.

**Figure 3:**
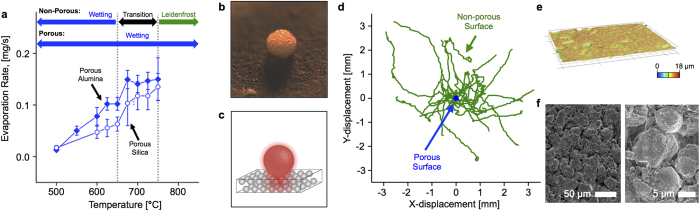
Structured Surfaces for Suppression of Cellulose Particle Liftoff. **a**. Cellulose particles (~220 μm) liquefy and evaporate at increasing rate as temperature increases on porous silica and alumina, with no measurable transition to film boiling. Error bars represent 95% confidence. **b,c**. Droplet of molten cellulose on porous alumina (image and diagram). See extended data for full video. **d**. Position of cellulose particles on polished silicon (green) and porous alumina (blue) indicate suppressed liftoff and motion (skittering) on porous materials. **e**. 3D profilometry of porous alumina indicates minimal surface roughness. **f**. Scanning electron micrograph reveals 1–5 μm macropores for sweeping product vapors away from particles.

Deviation from the Leidenfrost curve in Fig. 1a was observed when cellulose was pyrolyzed on porous pressed silica and alumina surfaces. Under these conditions, there was no observable transition to Leidenfrost behavior with increased temperature. As a result, the rate shown in Fig. 3a is nearly linear with temperature across the measured range (500–750 °C). Additionally, at temperatures below 675 °C, the observed heat transfer rates are lower compared with those from the polished silicon surface, indicating a lower solid-liquid heat transfer coefficient. However, at 775 °C, the heat transfer rate for the porous surfaces is higher than that observed for the polished surface. These results indicate that particle liftoff from vapor generation is completely inhibited across this temperature range, which agrees with previous work that suggests that vapors and gases from an evaporating liquid droplet penetrate into surface features, such as channels and macropores, thereby suppressing the Leidenfrost effect^11^,^12^,^22^,^23^,^24^,^25^,^26^. In Fig. 3b, a snapshot of a pyrolyzing cellulose particle on porous alumina at 750 °C, an expected Leidenfrost regime, shows no particle liftoff.

A common characteristic of droplets exhibiting the Leidenfrost effect is the ‘skittering’ or dancing motion across the surface, as is commonly observed with a superheated water droplet on metal surfaces. Traces of individual particle motion from high speed imaging for cellulose and polished and porous alumina surfaces are shown in Fig. 3d, with each trace starting at the plot origin. Significant particle motion was observed for the polished surface, with droplets moving in a generally consistent direction once initiated. However, in the case of the porous surfaces, almost no particle motion was observed (Figure S7^†^). Optical surface profilometry in Fig. 3e depicts flat surface topography, with peak to valley height of only 10–20 μm, indicating that the surface of porous alumina was relatively flat compared to the length scale of cellulose particles. SEM micrographs in Fig. 3f indicate visible macropores between alumina particles, giving support to the mechanism of gas flow through surface macropores for suppression of Leidenfrost behavior.

Dramatic difference in biopolymer behavior on heated, structured surfaces is integral to reactor design for utilization of natural resources such as biomass. Industrial scale pyrolysis reactions are commonly carried out in the presence of pressed silica and alumina based catalysts with macropores^27^,^28^, while the majority of fundamental pyrolysis studies are carried out on smooth metal surfaces^4^,^6^. The impact of porous structures and dramatic role of temperature on the de-wetting liftoff behavior of cellulose at high temperature allows for tuning to increase heat transfer and dramatically alter the throughput of biomass reactors, which are overall heat transfer limited systems. Alternatively, surface design to enhance cellulose particle liftoff has potential for separating biomass from product vapors, which could be used to separate inorganic material (ash such as SiO_2_) within biomass during reaction. Enhancement of biomass particle liftoff could reduce contact with solid surfaces, which serve as heterogeneous nucleation sites and lead to increased formation of molten cellulose aerosols. Design of structured surfaces and micro-ratchets for directing particle motion could also enable control of agglomeration for increased particle size, thereby controlling intermediate liquid lifetimes^29^. Further research into cellulose liftoff will allow for precise design of structured surfaces for control of the reactive Leidenfrost phenomenon.

## Experimental Methods

Cellulose impinged on polished and porous heated surfaces was imaged via integrated high-speed photography and microscopy. Cellulose liquid intermediate lifetimes were quantified and particle-surface interactions, such as wetting/de-wetting, contact angle, and surface spreading were measured via imaging. Particle motion was characterized during Leidenfrost film boiling via individual particle tracking.

Microcrystalline cellulose (Lattice NT-200, FMC-Biopolymer) was sieved to 200–250 microns. Silicon surfaces (1 cm × 1 cm × 500 μm, Valley Design Corp.) polished to <0.1 μ-inch were used as pyrolysis surfaces. Porous surfaces were pressed at 15 kpsi using γ-alumina particles (Strem Chemicals, P/N: 13-2525) and silica particles (Sigma Aldrich, P/N: S5505) to form cylindrical discs, which were calcined at 1000 °C for 5 hours. Grade 5.0 nitrogen (99.999% purity) was purchased from Middlesex Gases & Technologies.

For all experiments, a high-speed camera integrated with a microscope was used to image cellulose pyrolyzing on a temperature-controlled reactor stage. The Phantom eX2 high-speed camera was used to image particles at 1000 frames per second with an exposure time of ~100 μs. The camera was integrated with Olympus BX51 microscope 5x and 10x objective lenses (Olympus MPLN5x, MPLN10x). Lighting was provided from a mercury light source (Prior Lumen 200). The reactor stage was constructed using a cylindrical steel block with two integrated cartridge heaters and an optical pyrometer (Impac IGA-50-LO Plus) to form a feedback control loop with a PID temperature controller. The pyrometer was focused on each of the pyrolysis surfaces placed directly on the heated steel block to ensure accurate surface temperature measurement. Particles were imaged from above, at an angle by tilting optics, and from a 90-degree profile view using an aluminum-coated optical grade mirror (Edmund Optics, 49-405). Nitrogen flow was provided from all sides at 30 sccm using a ring-shaped diffuser.

To measure particle lifetime, cellulose particles were imaged from above as they pyrolyzed on each surface. A surface was placed on the reactor stage and allowed to reach thermal equilibrium, with the optical pyrometer aimed directly at the surface. Temperature measurements were performed without high-intensity lighting to reduce interference of microscope lighting on infrared temperature measurement. 5x microscope optics and high-intensity lighting focused at a 45-degree angle were used during cellulose pyrolysis imaging experiments. Between each particle experiment, nitrogen flow was turned off and residual char was burned off using a butane torch. The NIH program, ImageJ, was used to analyze image sequences by tracking apparent particle diameter as viewed from above with time. Evaporation rate was measured by manually tracing the area of the particle at which it was fully liquid and dividing this area by the time it took for the liquid to fully react. Cross-sectional area of pyrolyzing cellulose particles with time, as shown in Fig. 2a, was measured with time by performing uniform contrast and brightness adjustment and applying ImageJ particle counting feature. Particle movement, shown in Fig. 3d, was quantified using the NIH ImageJ Manual Tracking plugin to trace particle motion with time.

In order to visualize pyrolyzing droplets from the side as shown in Fig. 2b,c, 10x microscope optics were focused through a 45-degree mirror positioned adjacent to the heated surface. Images were backlit using the high-intensity light source. For angled visualization shown in Figs 1b,c, and 3b, 10x microscope optics were tilted to ~45 degrees and lighting was provided from a side angle.

Porous surfaces were characterized using optical surface profilometry and SEM (scanning electron microscopy). Surface profiles shown in Fig. 3e were obtained using a Keyence VHX-5000 digital microscope with 200x optics. SEM images in Fig. 3f were obtained using a JEOL JSM-6500F under 100x and 1000x magnification and 5 kV accelerator voltage.

## Additional Information

**How to cite this article**: Teixeira, A. R. *et al.* Reactive Liftoff of Crystalline Cellulose Particles. *Sci. Rep.* **5**, 11238; doi: 10.1038/srep11238 (2015).

## Supplementary Material

Supplementary Information

Supplementary Video 1

Supplementary Video 2

Supplementary Video 3
