# Supplementary material for: Reactive Liftoff of Crystalline Cellulose Particles
Source: Sci Rep. 2015 Jun 9;5:11238. doi: 10.1038/srep11238 (PMC4460903; doi:10.1038/srep11238)
Supplement: Supplementary Information [file srep11238-s1.pdf]

Supplementary Information:
Reactive Liftoff of Crystalline Cellulose Particles

Andrew R. Teixeira[1, ‡], Christoph Krumm[1,2, ‡], Katherine P. Vinter[1,2], Alex D. Paulsen[1,2], Cheng Zhu[1,2], Saurabh Maduskar[1,2], Kristeen E. Joseph[1,2], Katharine Greco[1], Michael Stelatto[1], Eric Davis[1], Brendon Vincent[1], Richard Hermann[2], Wieslaw Suszynski[2], Lanny D. Schmidt[2], Wei Fan[1], Jonathan P. Rothstein[1], Paul J. Dauenhauer[2,3,*]

[1]University of Massachusetts Amherst, Departments of Chemical and Mechanical Engineering, Amherst, MA, 01375

[2]University of Minnesota, Department of Chemical Engineering and Materials Science, Minneapolis, MN, 55455, USA.

[3]Catalysis Center for Energy Innovation, a U.S. Department of Energy – Energy Frontiers Research Center. University of Delaware, Newark, DE, 19716, USA.

[‡]Authors contributed equally.

*Corresponding Author: Paul J. Dauenhauer, hauer@umn.edu

Summary. The supplementary materials section includes additional details and data to support the conclusions presented in the main body of the manuscript. This section includes schematics of experimental setup and extended sets of frames from high-speed visualization. Quantitative dimensionless analysis is presented to contextualize the role of heat transfer in reactive cellulose droplets. Additionally, contact angles of reacting cellulose droplets are presented. Finally, data from all experiments, including all temperatures and surfaces, is presented on one plot for comparison.

Experimental Setup. The reactor stage and microscope setup described in the experimental section of the main paper was used in multiple configurations in order to visualize cellulose from the top, side, and at a 45-degree angle. The configuration presented in Figure S1a was used for the majority of experiments, including lifetime measurements, as well as overhead and 45-degree visualization. The angle of the microscope optics was altered in order to obtain the desired visualization angle. The configuration presented Figure S1b shows the experimental setup for measuring and visualizing the surface-cellulose interactions. The setup was backlit for contact angle measurements. Nitrogen flow to ensure an inert environment for pyrolysis was provided using a ring diffuser with holes along the inside, as shown in Figure S1c.

Determination of Evaporation Rate. The evaporation rate shown in Figure 1a and 3a of the main paper has units of milligrams of cellulose per second. This value is converted from the measured quantity of apparent change in area (mm^2/s) by multiplying by the cellulose particle density ($\rho = 1000\ kg/m^3$)[9] and the ratio of sphere volume divided by sphere cross-sectional area ($V_{sphere}/CA_{sphere} = (4/3)\pi R^3/\pi R^2$). Although this assumes that the initial particle is spherical, this is not a requirement for the entire particle lifetime because the particle area was not tracked with time – the evaporation rate is represented by the change from initial to final cross sectional area ($CA_F = 0$) over a measured lifetime.

Contact Angle Measurement. Additional experiments were conducted in order to identify the interactions between the cellulose droplets and each surface, shown in Figure S8. Room temperature contact angles were measured using water and ethylene glycol. The water contact

angle measured for the polished silicon was greater than those for the porous surfaces, suggesting that surface pores and roughness increase the wetting behavior. This trend was not present for reacting cellulose droplets, as all contact angles were approximately 60 degrees, except for that of cellulose on a polished surface at 750 °C, which fully de-wet. Despite large differences in heat transfer into the cellulose droplet between polished and porous surfaces at temperatures between 600-700 °C, the contact angles remained relatively constant.

Dimensionless Analysis. A comparison of timescale for heat convection and reaction kinetics is presented in the main body of the paper. The “Reacting Leidenfrost” quantity, ϕ_{RL}, compares the time constant for convection $\tau_{conv} = \rho C_p L_c/h$, with the time constant for reaction, $\tau_{rxn} = 1/k_{rxn}$. In these equations, ρ is the cellulose density (ρ = 1000 kg/m^3)[9], C_p is the cellulose heat capacity (C_p = 2300 J/Kg-K), h is the convective heat transfer coefficient between the surface and the cellulose particle (h = 10^4 W/m^2-K),[7] and k_{rxn} is the temperature dependent reaction rate.[17] L_c is the critical length scale taken to be R/3, the ratio of the sphere volume ($V = 4/3\pi R^3$) divided by the sphere surface area ($A = 4\pi R^2$). Comparison of τ_{conv} and τ_{rxn} reveals that kinetics are rate determining at temperatures below 500 °C. At temperatures between 500-700 °C, the kinetic and convective time constants are of similar magnitude, indicating competing rate determining phenomena. At high temperatures (T > 700 °C), the reaction rate is sufficiently high such that the particle evaporation rate is determined by convective heat transfer from the surface to the particle.

Surface Characterization. Characterization of the surface roughness of porous samples was conducted in addition to the optical profilometry and SEM presented in the main body of the paper. For the porous alumina surface, the primary average surface roughness was 1.71 μm and the waviness corrected average surface was 0.84 μm. Measurements were performed using a Veeco VCM optical profilometer with 20x optics and a scan distance of 243 μm. Waviness correction was performed using the algorithm provided by the Precision Engineering Division of NIST. A two-dimensional plot of three linear scans which were used for roughness and waviness calculations is shown in Figure S10.

Expanded frames. Expanded frames from each of the figures presented in the main body of the paper as well as frames from visualization not presented are shown in extended data figures. This includes angled visualization of cellulose on a polished silicon surface at various temperatures (Figure S3 and S4), angled visualization of cellulose on a porous alumina surface at various temperatures (Figures S5 and S6), and angled visualization of cellulose on a porous ZSM-5 surface at various temperatures (Figures S7 and S8).

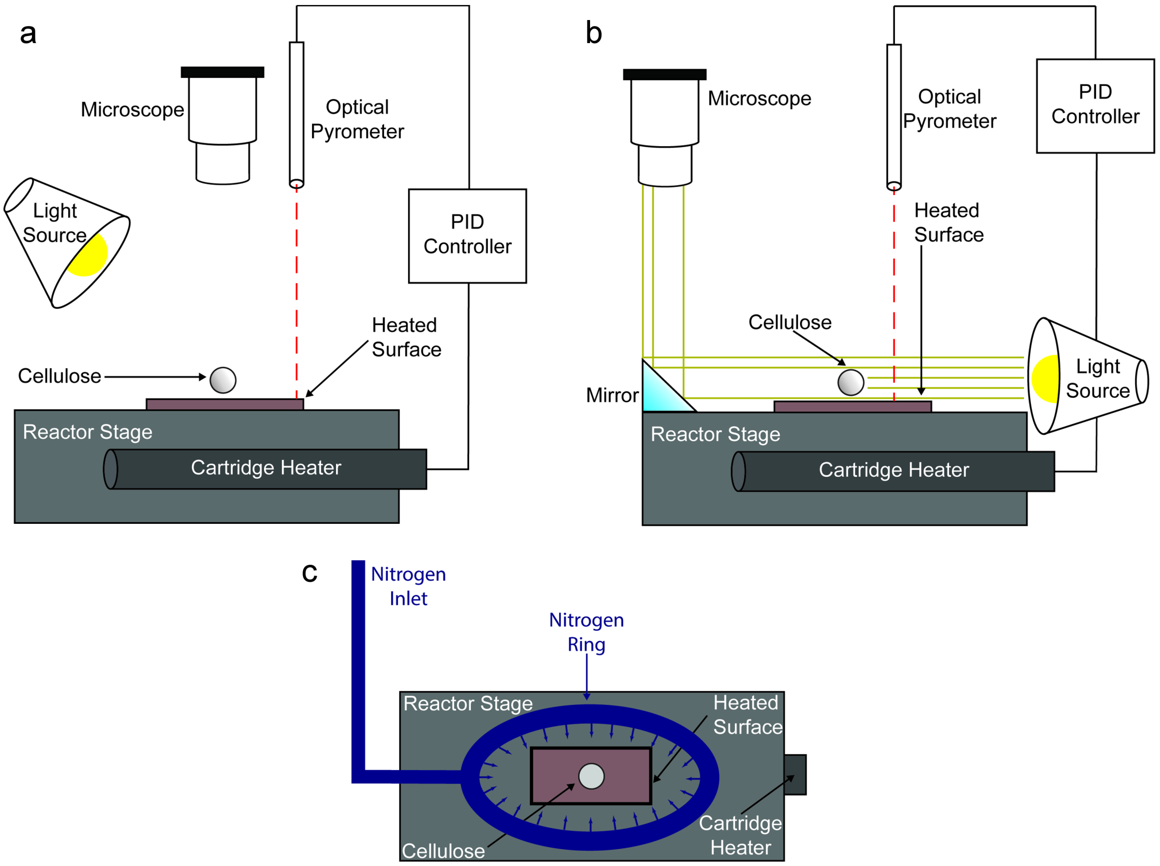

Figure S1 | Cellulose pyrolysis visualization experimental setup. A. The experimental setup for overhead visualization included a heated surface controlled via PID feedback with an optical pyrometer as well as a high power light source to illuminate the cellulose particles on the surface. **B.** Profile imaging of cellulose for visualization and contact angle measurements was performed with the same setup pictured in panel A, but offset to direct optics through a 45 degree mirror, with light provided from behind the particle. **C.** A top view of the reactor stage shows a diffuser ring for providing uniform nitrogen flow from all sides surrounding the heated surface and cellulose.

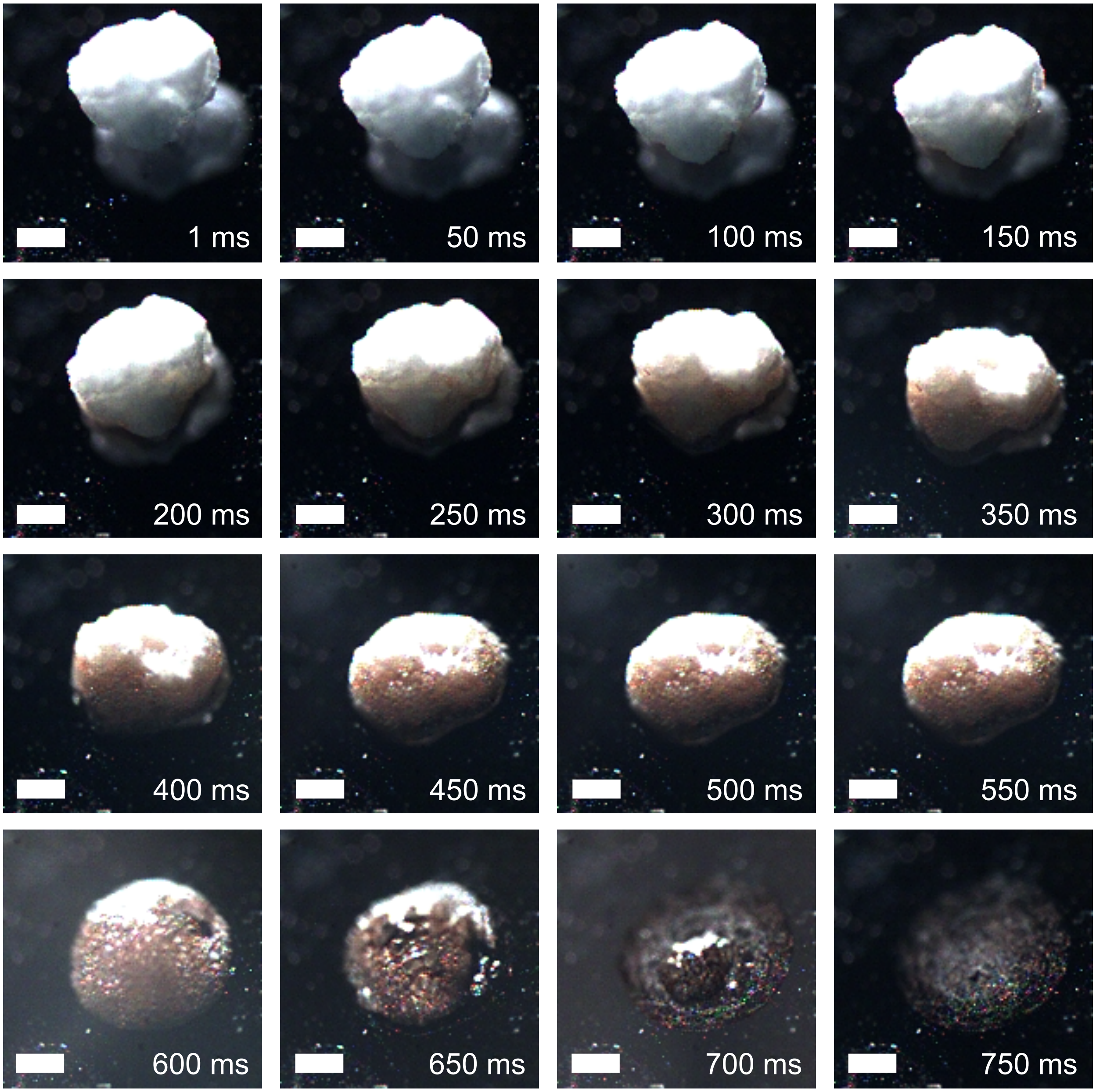

Figure S2 | Cellulose particle on polished silicon surface at 500 °C. Frames not presented in the main paper show an angled view of cellulose pyrolyzing on a polished silicon surface at 500 °C. Scale bars = 100 μm.

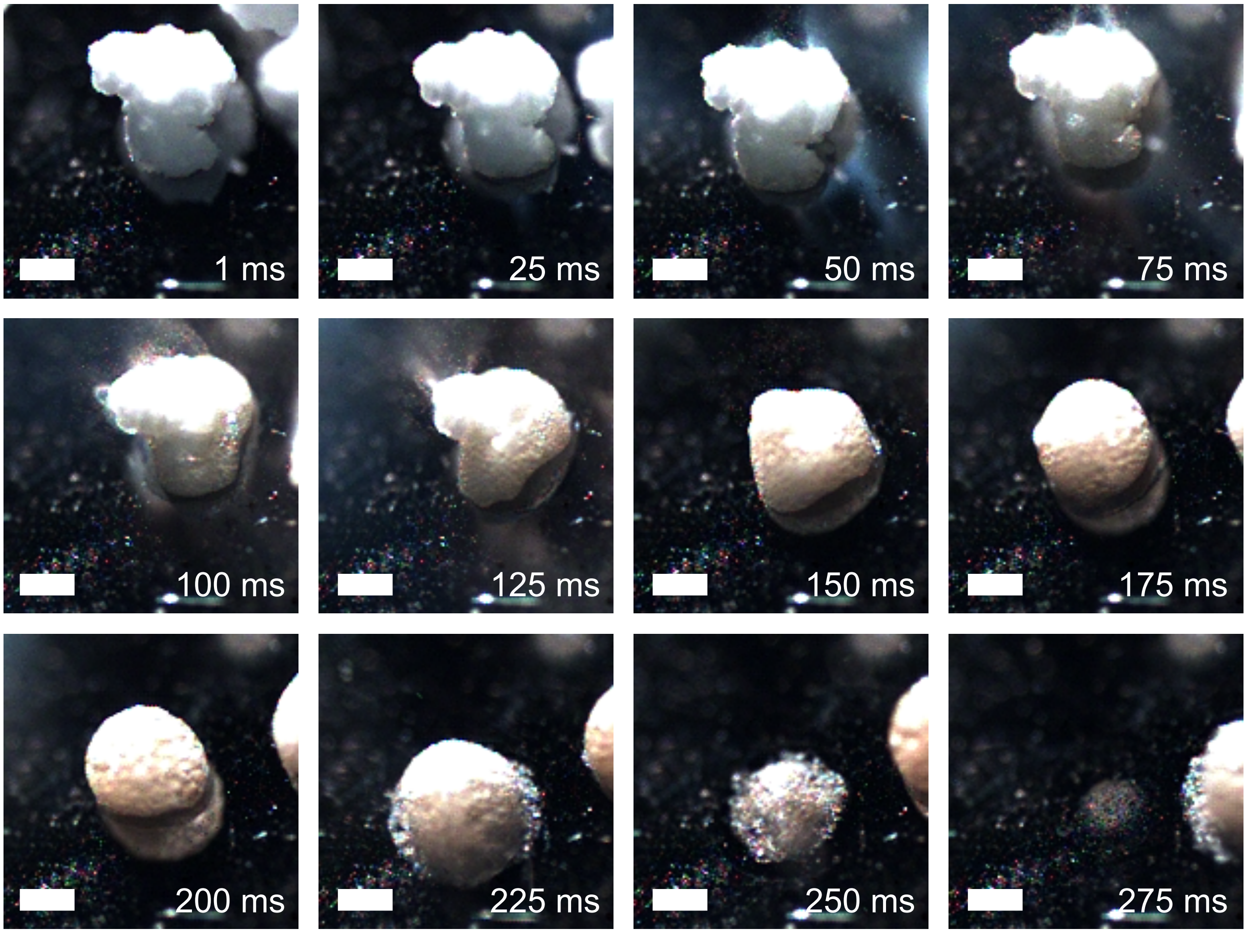

Figure S3 | Cellulose particle on polished silicon surface at 625 °C. Expanded frames of images shown in Figure 1b of the main paper show an angled view of cellulose pyrolyzing on a polished silicon surface at 625 °C. Scale bars = 100 μm.

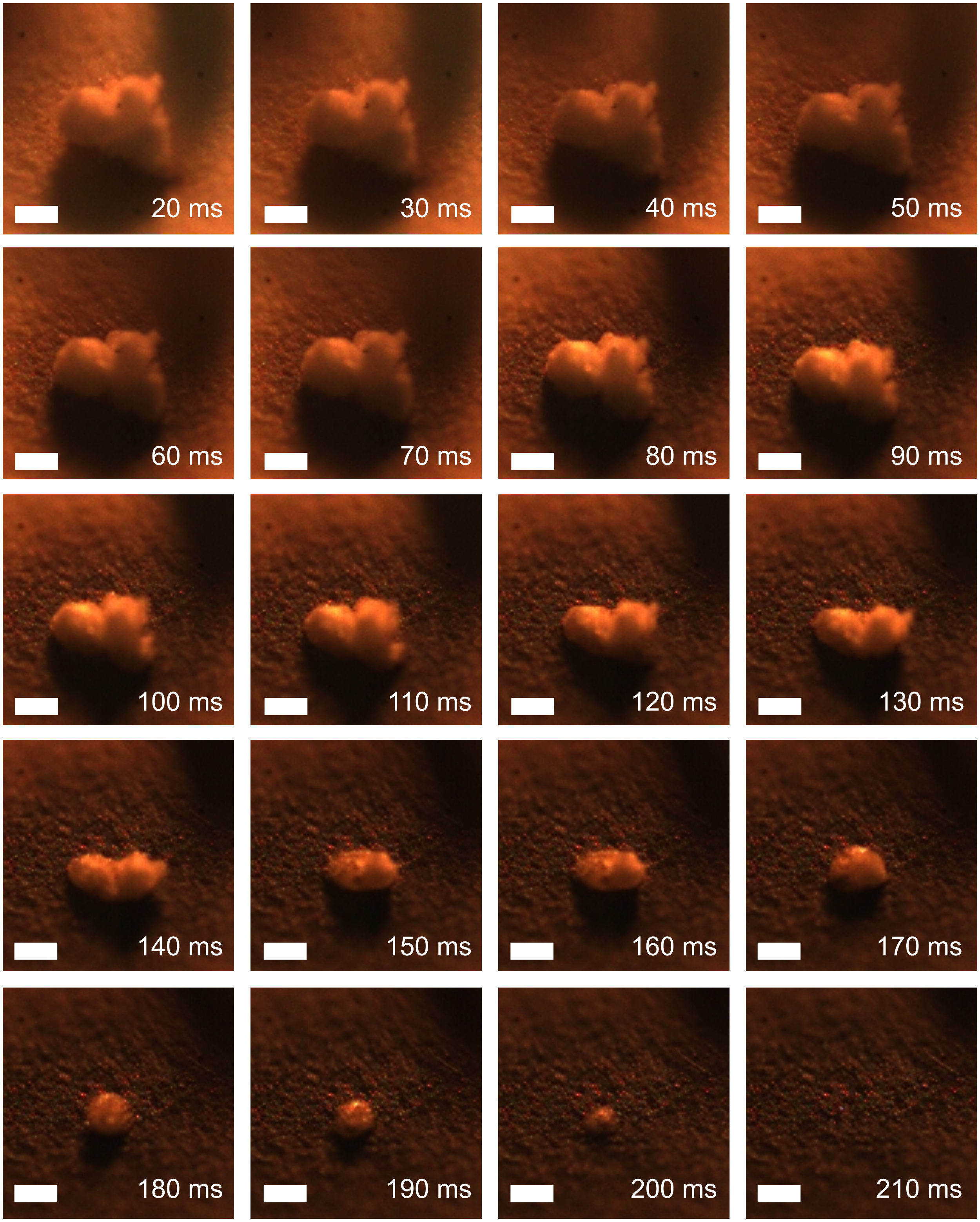

Figure S4 | Cellulose particle on pressed alumina porous surface at 625 °C. Frames not presented in the main paper show an angled view of cellulose pyrolyzing on a porous alumina surface at 625 °C. Scale bars = 100 µm.

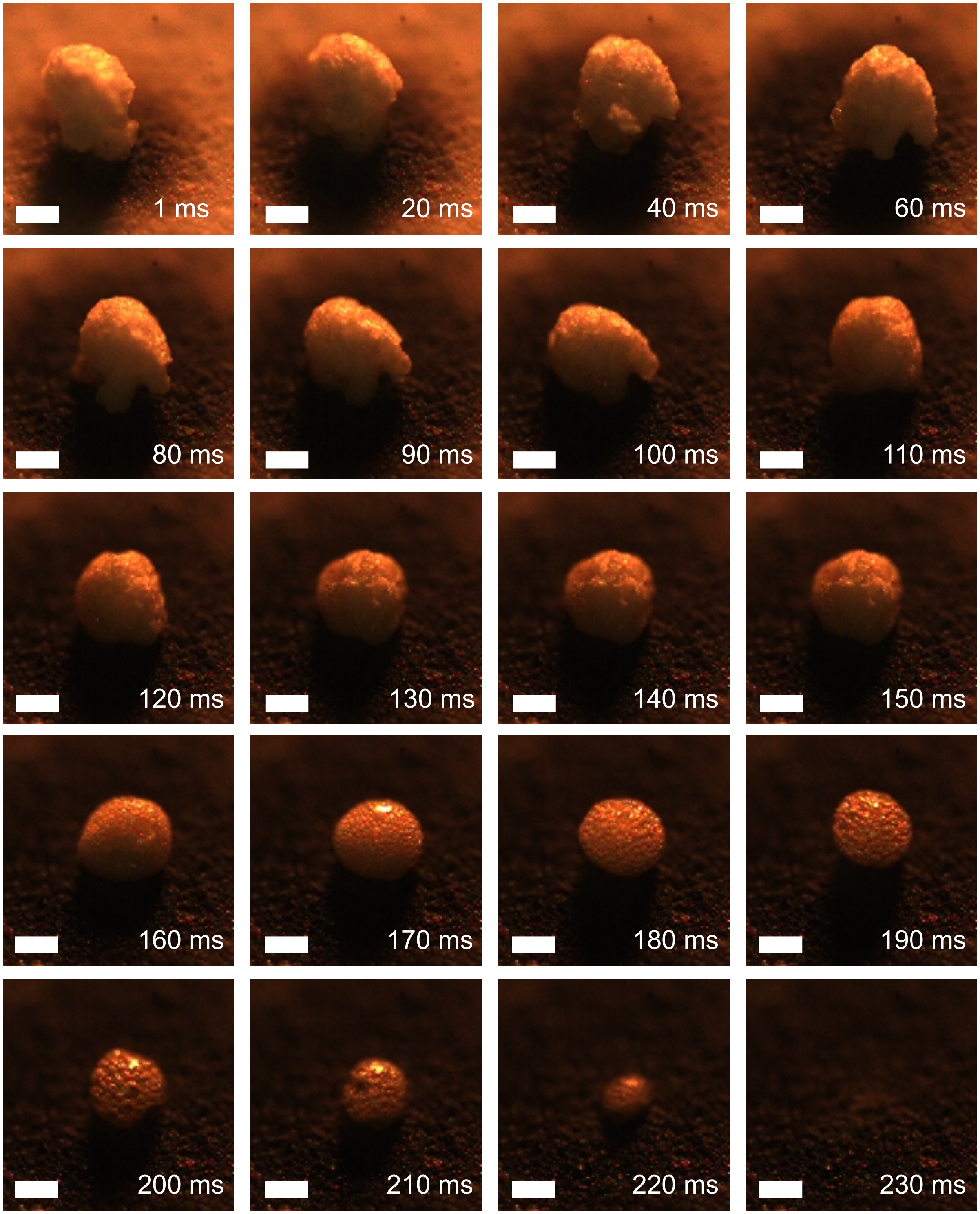

Figure S5 | Cellulose particle on pressed alumina porous surface at 750 °C. Expanded frames of the image shown in Figure 3b of the main paper show an angled view of cellulose pyrolyzing on a polished silicon surface at 750 °C. Scale bars = 100 µm.

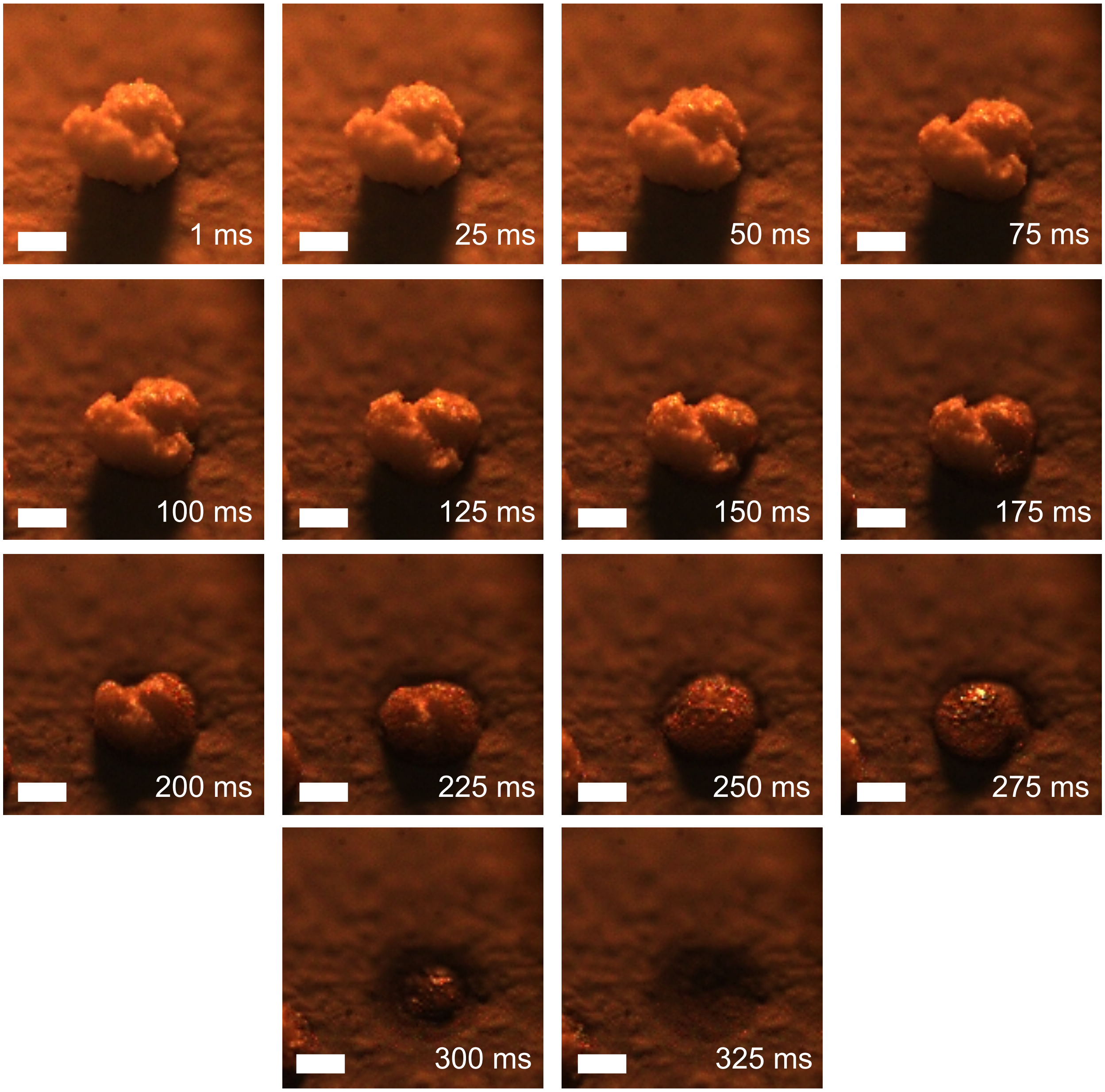

Figure S6 | Cellulose particle on pressed ZSM-5 porous surface at 625 °C. Frames not presented in the main paper show an angled view of cellulose pyrolyzing on a porous ZSM-5 surface at 625 °C. Scale bars = 100 µm.

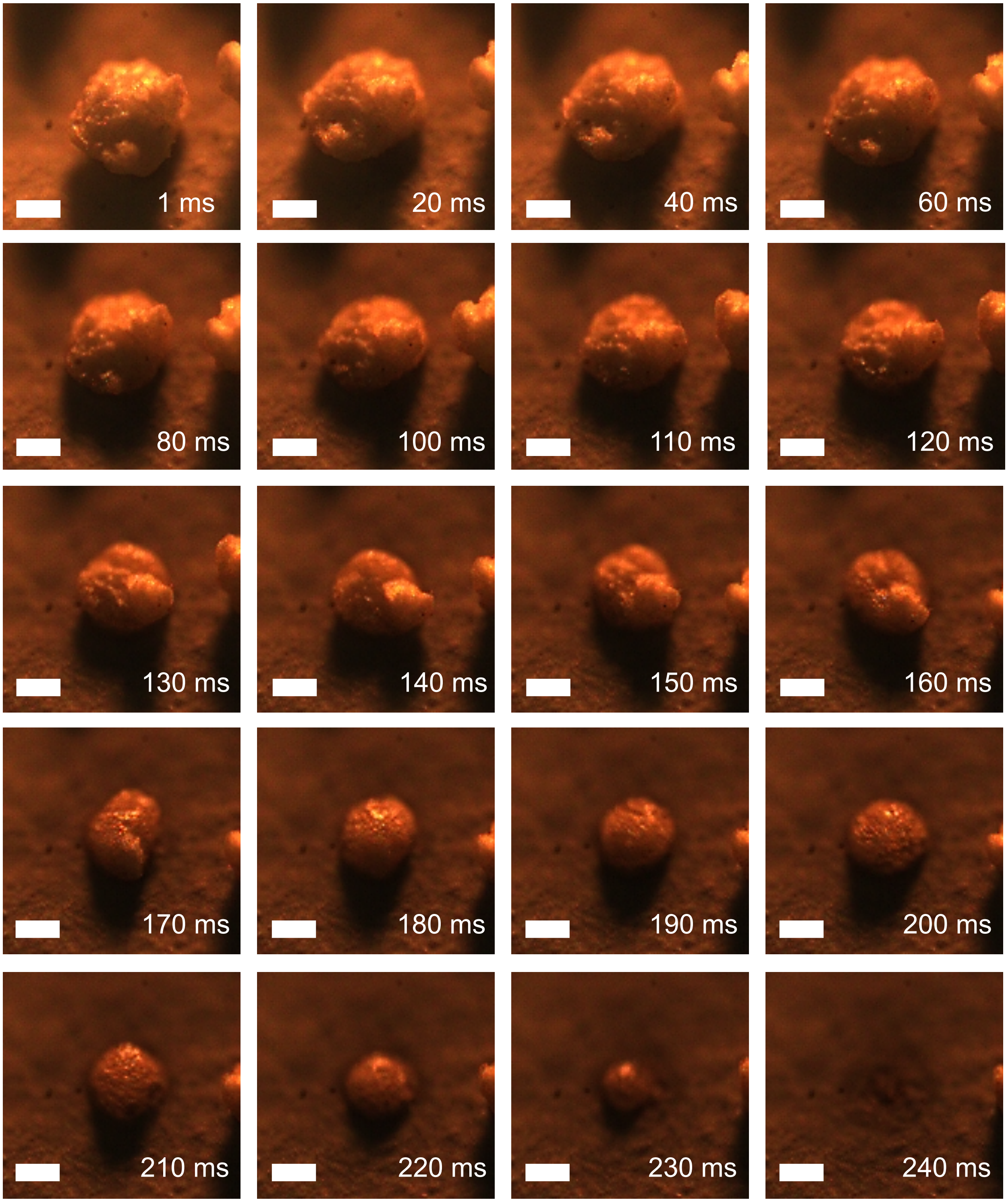

Figure S7 | Cellulose particle on pressed ZSM-5 porous surface at 750 °C. Frames not presented in the main paper show an angled view of cellulose pyrolyzing on a porous ZSM-5 surface at 750 °C. Scale bars = 100 µm.

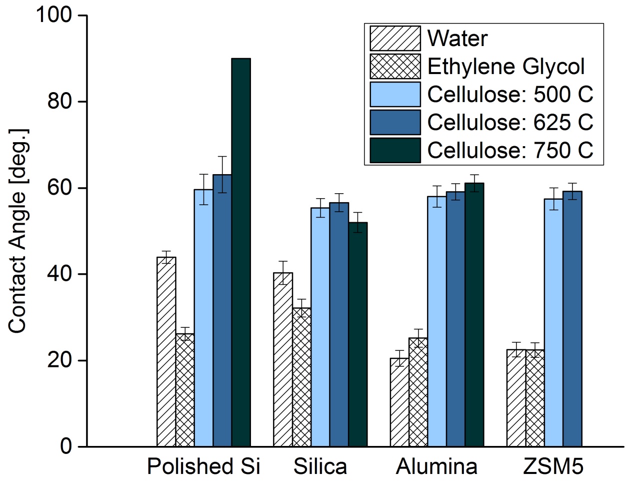

Figure S8 | Contact angle measurements for surfaces used in experiments. Contact angle measurements vary between surfaces for water and ethylene glycol at room temperature, but remain relatively constant for cellulose reacting at various temperatures. Contact angles for particles in the Leidenfrost regime are reported as 180°, as they fully de-wet the surface.

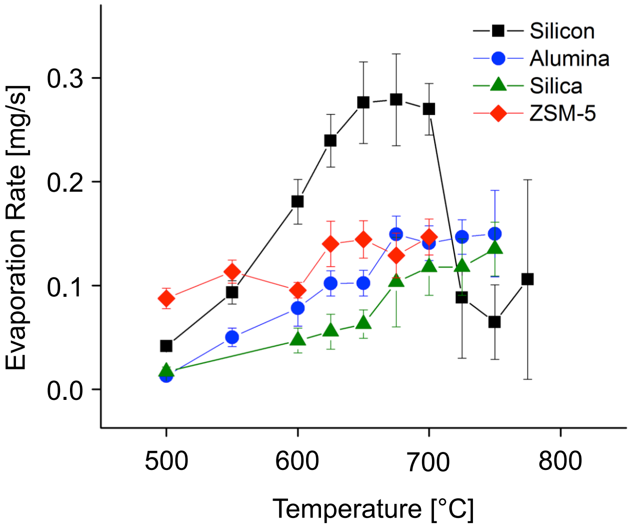

Figure S9 | Lifetime Data versus temperature for all surfaces. The overlaid evaporation rate data for all surfaces at all temperatures shows suppression of the Leidenfrost effect for all porous surfaces. Between 650-750 °C, a 2-10 fold difference in heat transfer from the surface to the particle is observed between the polished and porous surfaces. Error bars represent 95% confidence.

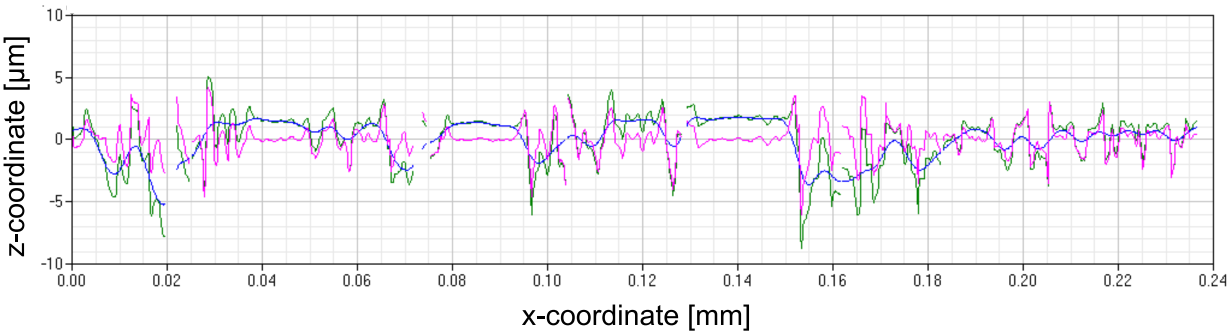

Figure S10 | Two-dimensional profilometry of porous alumina. Three linear profiles of the surface of a porous alumina disc used as a pyrolysis surface show a peak to valley height of approximately 10 µm, with a waviness corrected surface roughness of 0.84 µm.

Supplementary Video 1 | Angled view of cellulose particle on polished silicon surface at 625 °C. A ~220 micron cellulose particle impinging on a polished surface exhibits strong wetting behavior as the solid cellulose reacts to form a melt and evaporates.

Supplementary Video 2 | Angled view of cellulose particle on porous alumina surface at 750 °C. A ~220 micron cellulose particle impinging on a porous surface does not lift off from the surface and moves very little, suggesting that the anticipated cellulose Leidenfrost effect is suppressed on porous surfaces at high temperatures.

Supplementary Video 3 | Profile view of cellulose particle on polished surface at 625 °C. A ~220 micron cellulose particle impinging on a polished surface exhibits strong wetting behavior as the solid cellulose reacts to form a melt and evaporates.
